# The working alliance in stuttering treatment: a neglected variable?

**DOI:** 10.1111/1460-6984.12465

**Published:** 2019-03-13

**Authors:** Hilda Sønsterud, Melanie Kirmess, Kirsten Howells, David Ward, Kristin Billaud Feragen, Margrethe Seeger Halvorsen

**Affiliations:** ^1^ Department of Psychology University of Oslo Oslo Norway; ^2^ Statped Department of Speech and Language Disorders Oslo Norway; ^3^ Department of Special Needs Education University of Oslo Oslo Norway; ^4^ Sunnaas Rehabilitation Hospital Norway; ^5^ Independent scholar Wilmslow UK; ^6^ University of Reading Speech Research Laboratory Reading UK; ^7^ Oslo University Hospital Centre for Rare Disorders Oslo Norway

**Keywords:** stuttering, stuttering treatment, client–clinician relationship, working alliance, motivation, treatment outcome

## Abstract

**Background:**

Multiple factors can influence the working alliance and treatment outcome in speech and language therapy. The ‘working alliance’ is an important concept in treatment and can be described as the degree to which a treatment dyad is engaged in collaborative, purposive work. To date, relatively little attention has been paid to this concept within speech and language treatment in general, and within stuttering treatment research in particular.

**Aims:**

To investigate the role of the working alliance within stuttering treatment, and to evaluate whether the quality of the working alliance correlated with clients’ concept of motivation and treatment outcomes 6 months post‐therapy.

**Methods & Procedures:**

Eighteen adults (21‐61 years) participated in this multiple single‐case treatment study, with treatment facilitated by an experienced speech and language therapist. The working alliance was investigated using the Working Alliance Inventory—Short Version Revised (WAI‐SR), an Extended version of the Client Preferences for Stuttering Treatment (CPST‐E), the Overall Assessment of Speakers’ Experience of Stuttering—Adult version (OASES‐A), the Wright & Ayre Stuttering Self‐Rating Profile (WASSP) and the Hospital Anxiety and Depression Scale (HADS).

**Outcomes & Results:**

Analyses demonstrated significant associations between the working alliance and client motivation (r = 0.781) and treatment outcomes (r = 0.644) 6 months post‐treatment. The association between client‐led goals and therapy tasks appeared particularly important.

**Conclusions & Implications:**

: The working alliance between speech and language therapists and persons who stutter matters. Within the alliance, the level of client–clinician agreement on treatment goals and therapy tasks may be of greater importance than the bond between client and clinician. Further research with greater numbers of participants is warranted.

**What this paper adds**

**What is already known on the subject**

The concept of the working alliance has its roots in psychodynamic theory, and this concept represents a proactive collaboration of clients and therapists across treatment sessions. Within that literature, it has been demonstrated that a client's opinion of treatment as effective or ineffective is influenced by their experience of the collaborative process in the clinic. Treatment evaluations should therefore incorporate evaluation of the client‐clinician relationship, particularly from the perspective of the client. There has been some related work within the speech and language therapy literature, but to date, relatively little attention has been paid to the working alliance within clinical work and research associated with management for stuttering.

**What this paper adds to existing knowledge**

As far as the authors are aware, this is the first published study to have investigated the working alliance in relation to a stuttering management trial. Results indicate that the working alliance is highly relevant in the evaluation of treatment outcomes. The study indicates that the working alliance between a speech and language therapist and a person who stutters matters, providing support for similar findings within other fields of speech and language therapy. Based on Bordin's model of the working alliance, which includes the dimensions of therapy goals, therapy tasks and the bond between client and clinician, the findings indicate that the dimensions of goals and tasks were particularly relevant. The clients’ motivation for treatment, and agreement regarding meaningful tasks for achieving change, may become important predictors of successful treatment outcomes.

**What are the potential or actual clinical implications of this work?**

The study indicates that speech and language therapists should be aware of the importance of the working alliance within treatment. Relevant and specific quantitative and qualitative assessments for measuring the therapeutic alliance, particularly from the client's perspective, are needed to explore this concept in more detail. The Working Alliance Inventory—Short Revised version (WAI‐SR) is one such tool that can be used to evaluate elements of this relationship. The working alliance includes elements such as a shared understanding of treatments goals, agreement regarding treatment tasks, and the bond between the client and the clinician.

## Background

Stuttering's variability and unpredictability suggest that it can be regarded as a complex disorder (Packman and Kuhn 2009, Ward 2018), and flexible therapeutic approaches are needed to deal with this complexity (Baxter *et al*. 2015).

### Causal complexity in clinical practice: a non‐linear interaction

There are several models for explaining different factors that might lead to therapeutic change, and some are outlined in this paper (Cartwright and Hardie 2012, Lambert 2013, Wampold 2015). When an intervention is implemented, outcomes will therefore be affected not only by the intervention itself but also by other factors. In the field of psychology, Lambert (2013) has summarized outcome research and grouped the factors of successful therapy into four areas and based on the literature roughly estimated percentages of change in clients as a function of therapeutic factors. The relative influence of these factors was estimated as follows: client/life 40% (qualities of the client or the environment), common factors 30% (empathy and the therapeutic relationships), expectancy 15% (client's expectation of help or belief in the therapy), and techniques 15% (factors unique to specific therapies and tailored to management of specific problems) respectively. Based on his valuations, client factors exert the greatest influence, followed by common factors, the techniques employed and expectations regarding outcome. Other models, such as the Common Therapeutic Change Principles (Goldfried 1980) and the Contextual Model (Wampold 2015), seem to parallel to some extent the Lambert's (2013) pie chart, and are models presented in the work of, for example, Manning (2010b) and Plexico *et al*. (2010).

The Contextual Model described by Wampold (2015) is grounded in the social sciences and takes into account social healing aspects of psychotherapy: ‘The basic premise of the model is that the benefits of psychotherapy accrue through social processes and that the relationship, broadly defined, is the bedrock of psychotherapy effectiveness’ (50). The model explicates three main pathways that engender change through therapy: (1) a real relationship between the client and clinician, (2) the creation of expectation through treatment rationale and (3) therapeutic tasks and actions that correspond with that treatment rationale. According to Wampold, the clinician and client have to establish an initial bond ‘before the three pathways can be employed’ (53–54).

In contrast to Lambert's (2013) pie chart, which estimates the degree of influence exerted by different factors on treatment outcomes, the Contextual Model (Wampold 2015) does not indicate the extent of the influence exerted by different factors. This provides a more flexible framework, as it allows for the possibility that the relative influence of different factors may vary dependent on several elements. Such elements include among other elements, the disorder, the contextual variables, and the within and between variables of both the clinician and the client (Low 2017, Mumford 2011).

### The concept of the working alliance

The concept of the clinical alliance has its roots in psychodynamic theory, and commands considerable attention in the psychotherapy literature (Bordin 1979, Flückiger *et al*. 2018, Horvath *et al*. 2011, Wampold 2015). Treatment outcomes and an individual's experience of treatment as effective or ineffective may incorporate evaluation of the client‐clinician relationship. It was Bordin (1979) who first named this relationship the ‘working alliance’, describing it as the degree to which the therapy dyad is engaged in collaborative, purposive work. The working alliance is further described as the healthy, trusting aspect of the client–clinician relationship (Bordin 1979, Hatcher and Gillaspy 2006, Horvath and Greenberg 1989) and may be influenced by factors such as the individual's faith in the treatment process, and their expectations regarding a positive or negative outcome (Manning 2010b, Plexico *et al*. 2005, 2010). According to Flückiger *et al*. (2018), ‘The alliance represents a proactive collaboration of clients and therapists across sessions and in moment‐to‐moment interactions’ (330). Bordin and others suggest that the working alliance has its foundation in the following three processes: (1) the emotional *bond* between the client and clinician, (2) the extent to which the client and clinician agree on the *goal* of treatment and (3) the extent to which the client and clinician consider the treatment tasks as relevant (*task*) (Bordin 1979, Hatcher and Gillaspy 2006, Horvath *et al*. 2011).

The potential impact of the client–clinician relationship is also acknowledged within communities of people who stutter. For example, the podcast and online community StutterTalk© recently published a position statement that includes the paragraph: ‘As the therapeutic relationship is built upon trust and understanding, let yourself “shop around.” If you don't feel comfortable with the first therapist you meet, visit with another’ (http://stuttertalk.com/stuttertalk-position-statement-on-self-help-and-speech-therapy-for-people-who-stutter/, 21 February 2018).

Although the relationship between the working alliance and treatment outcomes has received consistent support across studies within psychotherapy (Del Re *et al*. 2012, Flückiger *et al*. 2018, Wampold 2015), the literature regarding the role of the working alliance within speech and language therapy (SLT) is relatively limited to date (Bright *et al*. 2011, Caughter and Dunsmuir 2017, Fourie 2009, Lawton *et al*. 2018a, 2018b). As with the field of physiotherapy (Kayes and McPherson 2012, Miciak *et al*. 2018), SLT has borrowed theory from psychotherapy to inform the research and practice. Within stuttering treatment research, the relationship between the working alliance and treatment outcomes have received little attention, with the exception of the work of Manning (2010b) and Plexico *et al*. (2010). In summary, although many SLTs agree that the client–clinician relationship matters in clinical practice and research (Manning 2010a, Shapiro 2011, Van Riper 1973, Ward 2018, Zebrowski and Kelly 2002), there are, as far as we are aware, few studies investigating the working alliance within the field of fluency disorders, and in particular from the client's perspective. Clinical experiences suggest this may in part be due to a lack of time and awareness amongst SLTs regarding relevant quantitative and qualitative assessments for measuring the client–clinician relationship.

We assume that, as with what is evidenced in psychotherapeutic or physiotherapeutic practice, clinical judgments and a stable and positive working alliance contribute to successful outcomes also in SLT. The correlation between working alliance and treatment outcomes has been examined meta‐analytically in psychotherapy several times, with overall correlations varying only slightly (*r* = .21–.29) (Baldwin *et al*. 2007, Flückiger *et al*. 2018, Horvath *et al*. 2011). The research confirms that the working alliance is an important contributor to treatment outcomes, but it also indicates that other factors are influential.

Based on meta‐analysis (Del Re *et al*. 2012, Flückiger *et al*. 2018), it appears that therapist variability in the working alliance potentially has a greater influence on treatment outcomes than the clients’ variability. According to the researchers, these results suggest that some therapists develop stronger alliances with their clients irrespective of diagnosis, and as a result; their clients ‘do better at the conclusion of therapy’ (Del Re *et al*. 2012: 648).

We believe there is a need to consider the working alliance as an evidence‐based component within stuttering therapy, including the extent to which the alliance alters or remains stable during the treatment period. This is, perhaps, particularly relevant to adults who stutter, where goal‐directed management requires high levels of clinical competence, and calls for more individualized treatment procedures (Manning 2010b, Plexico *et al*. 2010, Ward 2018).

### The working alliance in stuttering treatment

To our knowledge, previous stuttering therapy studies have not fully considered the range of factors that may influence treatment outcomes. The present study aims to address this by considering the interaction between the client's concept of motivation, the working alliance, the rationale for individualized cognitive restructuring and physical adjustments, and the subjective experience of how the resulting changes influence the nuances of the person's life. In this way, we intend to incorporate various elements of the Contextual Model into our evaluations.

Consistent with the work of Manning and colleagues (Manning 2010a, 2010b, Plexico *et al*. 2010), we contend that the Common Therapeutic Change Principles and the Contextual Model can be transferred to, and consciously used in, speech therapy in general, and stuttering treatment in particular. Plexico *et al*. (2010) highlighted three aspects as essential components of an effective stuttering treatment for adults: (1) clinician's understanding of stuttering, (2) a positive client–clinician alliance and (3) the clinician being knowledgeable about stuttering and its treatment.

Some researchers working within the discipline of psychotherapy have found that the clinician's interpersonal style influences both the quality of the alliance and the therapeutic process (Anderson *et al*. 2009, Nissen‐Lie *et al*. 2013, Oddli and Halvorsen 2014). We would also include the clinician's interpersonal style and the continued relevance of the flexibility, honesty, respect, trustworthiness, confidence, warmth, interest and openness highlighted by authors such as Ackerman and Hilsenroth (2003) or Van Riper (1973) for SLTs working with individuals who stutter. Those aspects mirror, for example, the work of Miciak *et al*. (2018), who identified four main conditions necessary for establishing a therapeutic relationship: being present, receptive, genuine and committed. According to the authors, these conditions in conjunction with applying communication skills, represent the intentions and attitudes of both the clinician and client.

Herder *et al*. (2006) speculate that the critical element(s) for successful stuttering intervention might not lie within the intervention itself, but rather on two major conditions; the intervention strategy and the specific or combined characteristics of individuals who stutter. In this way, Herder *et al*. emphasize the influence of client characteristics rather than clinician characteristics in relation to treatment. Several authors have drawn attention to client features in relation to stuttering treatment, including psychological distress in general, and anxiety in particular (Craig and Tran 2014, Iverach *et al*. 2017, Iverach and Rapee 2014).

In line with the work of Baxter *et al*. (2015), when questioning why particular interventions appear to work better with particular clients, Manning (2010b) states ‘regardless of the particular treatment approach, factors such as the working alliance between the client and the clinician, and clinician allegiance to the treatment protocol are important’ (314). The working alliance includes a shared understanding of treatment goals and the relevance of the therapeutic tasks to these goals. Although these specific elements will be investigated in more detail in this study, we regard the various perspectives outlined as relevant, contending that multifactorial and contextual understanding, including understanding and acknowledgement of individual preferences and goals defined before treatment, facilitate successful stuttering therapy (Manning 2010a, Plexico *et al*. 2010, Ward 2018). Within the present study, we apply Bordin's (1979) model of the working alliance. This model is easy to administer, and it allows researchers to study the relationship between the alliance and outcome within a number of therapies (Baldwin *et al*. 2007, Munder *et al*. 2010).

### The clients’ concept of motivation

The concept of motivation give people actions a direction for achieving a goal. Different theories have been proposed to explain motivation, and the degree of motivation is considered as one important reason that inspires a person to ‘move forward’ and may influence the treatment process and outcome (Cox and Klinger 2004). In this study, motivation was regarded broadly, in which the term ‘motivation’ was referring to goal‐related processes comprising both psychological and social factors. Particularly, we recognize the range of client and clinician characteristics which interact in the client–clinician alliance. One characteristic that has the potential to influence the treatment process and outcome is the client's degree of motivation. We base our concept of motivation on Seo *et al*.’s (2010) ‘work motivation’ model. Briefly, Seo *et al*. identify the following three components to motivation: generative orientation (characterized by active engagement to achieve anticipated positive outcomes), effort (which refers to how much time and energy a person devotes to selecting and executing action to complete a given task), and persistence (maintaining an initially chosen course of action over time).

### Aims

As part of a wider ranging study of individualized stuttering management tailored to the participants’ personal goals and preferences (Sønsterud 2015), the present study studied the roles of the working alliance more closely. This aim was to investigate the role of the working alliance within stuttering treatment and to evaluate whether the quality of the working alliance correlated with clients’ motivation and treatment outcome 6 months post‐therapy.

## Methods and procedures

### Research design: multiple single case design (MSCD)

In this study, an ABA, multiple single‐case design methodology (Tate *et al*. 2016) was used to collect data on each participant pre‐therapy, during the therapy sessions, and at 6 months post‐therapy. Within this framework, we were able to evaluate the working alliance, as well as behavioural, social and emotional aspects related to stuttering over time. This design facilitates consideration of the participants’ subjective experience of the working alliance, the concept of motivation and stuttering management outcome.

### Participants and recruitment

Twenty‐nine adults who stutter were initially recruited. Owing to practical challenges related to long‐distance travel in conjunction with treatment and testing commitments, eight responders were excluded, leaving 21 adults for the pre‐treatment phase. Of these, one participant (participant 8) was excluded by the SLT due to a co‐diagnosis of cluttering, and two further participants withdrew during the pre‐treatment phase due to challenging work and health circumstances. This left a total of 18 adult participants (*n =* 18), with the final treatment cohort thus comprising 15 men and three women aged 21–61 years, with a mean age of 35.8 years. Based on the participants’ responses on OASES‐A (Yaruss and Quesal 2006), an experienced SLT diagnosed all participants with moderate to severe stuttering before enrolment in the study. Mean total impact stuttering score was 2.80 (SD = 0.61), indicating a moderate to moderate‐severe impact rating. Demographics and other relevant background variables are presented in table 1.

**Table 1:** Baseline characteristics of the 18 participants

Background variables	% (*n*)
Male gender	83.3 (15)
Stuttering in the family	44.4 (8)
Higher academic education ≥ 3 years	33.3 (6)
Vocational rehabilitation	11.1 (2)
Received SLT treatment as children	61.1 (11)
Received SLT treatment as adults	16.7 (3)
No previous stuttering treatment at all	22.2 (4)

Note: Data are percentages and frequency (*n*).

The participants’ self‐reported severity rating scores included the mean score of the overall impact of stuttering, and the four subscales, presented in table 2. The Norwegian reference group (*n =* 62) was used (Nordbø *et al*. 2018) to compare means of the five impact scores (overall score and four subscores) with results from the present study. As seen in table 2, the Norwegian norms are generally lower than the mean scores for the participants in this study. This suggests that the participants from the present study, on a group level at pre‐treatment, had a slightly greater degree of negative impact associated with stuttering when compared with the reference group.

**Table 2:** Results of OASES‐A: impact and subscale scores before treatment compared with the Norwegian norms

Study sample (*n* = 18); reference group (*n* = 62)				
Sections OASES‐A	Mean	SD	Mean	SD
Overall stuttering impact	2.83	0.62	2.61	0.61
General information	3.06	0.45	2.66	0.51
Reactions	2.95	0.68	2.77	0.63
Communication	2.76	0.80	2.66	0.73
Quality of life	2.61	0.76	2.31	0.82

Note: SD, standard deviation.

### Clinical setting and data‐collection procedures

The clinical setting was Statped,^1^ the Department of Speech and Language Disorders, in Oslo, Norway. The participants underwent a 6 weeks of pre‐treatment phase in which the first evaluation took place during the first week within the clinic setting and was facilitated by the SLT. Evaluation included validated and internationally recognized measurements, examining stuttering, reactions to stuttering, communication and quality of life. With the aim of measuring stuttering variance over time, each participant was instructed to evaluate their own stuttering severity on a weekly basis outside the clinic visits. After 6 weeks, the participant's stuttering was measured again within the clinic setting. The intervention started immediately after the pre‐treatment phase and consisted of an 8‐week treatment period of four treatment sessions (scheduled during weeks 1, 2, 4 and 8 of the treatment phase). The average duration of each treatment session was 2.5 h.

The intervention approach entitled ‘Minding the body in speech’ was carried out by an experienced SLT and based on individualized treatment goals developed in consultation with each participant. The intervention was holistic, client‐centred and was based around five areas of focus: (1) awareness of body tension and posture, (2) awareness of breath support in speech production, (3) awareness in speech production to promote easier voicing, (4) awareness of acceptance, and mindfulness‐based strategies and (5) awareness of presentation skills. The relative prominence of the specific elements within each of the five areas of focus was adjusted according to the needs of each individual. Participants were expected to work independently between management sessions. Evaluations of stuttering, reactions to stuttering, communication and quality of life were repeated 6 months post‐intervention. Note that the wider treatment study employed a greater range of assessments, encompassing both physical and psychological factors, with outcomes for social, emotional and behavioural characteristics. In the present study, only a subset of these measurements was included.

After the third (T1, second treatment session), fifth (T2, fourth treatment session) and sixth (T3, 1 month post‐treatment) face‐to‐face meeting, paper‐and‐pen versions of the WAI‐SR were handed out. The participants received brief information regarding the WAI‐SR, stating that the questions contained therein related to their view of the collaboration with the SLT, and that the purpose of including the WAI‐SR in this study was to evaluate its use within SLT. All participants completed the three evaluations of the working alliance during a period of 3 months (second and fourth treatment sessions, and at 1 month follow‐up). As this evaluation was part of a stuttering management study, the clinician was blinded to the participants’ responses, which were submitted in sealed envelopes, and all participants were assured that the SLT would not see their responses until 6 months after the treatment period was complete. After the end of treatment, and when the 6‐month follow‐up was completed, a professional (a qualified lawyer and SLT) witnessed the opening of the envelopes by the SLT (researcher).

### Measures and materials

A view that has gained wide support is that the client is best placed to evaluate many aspects of clinical change; alongside clinic‐based measures, treatment outcomes therefore include self‐evaluation by the client (Bothe and Richardson 2011, Ingham *et al*. 2012, Manning 2010b). Our aim was to employ relevant measures, considering the participants’ aims and including reliable and valid measures of speech behaviour, cognition and emotional state, as well as the ability to communicate in a variety of social and professional situations in daily life. To evaluate the working alliance, the study incorporated measures of the value the participants placed on the therapy goals, therapeutic bond, and therapy tasks. In addition, an Extended version of the form Client Preferences for Stuttering Treatment (CPST‐E) (McCauley and Guitar 2010) was included, collecting both qualitative and quantitative data, and requiring the participants to describe their personal goals and priorities before treatment.

### Assessment of the working alliance (WAI‐SR)

The Working Alliance Inventory (WAI) is a self‐report instrument used to measure the strength and quality of the relationship between client and clinician (the participant and the SLT). The original version of the WAI (Horvath and Greenberg 1989) has 36 items spread across three subscales: *bond*, *goal* and *task*. In the present study, the quality of these elements of the working alliance was evaluated using the short version of the Working Alliance Inventory—Clients’ ratings (WAI‐SR) (Hatcher and Gillaspy 2006).

This short version includes 12 items scored on a seven‐point Likert scale, with high values indicating a strong therapeutic alliance. The available scores for each of the three subscales (*bond*, *goal* and *task*) range from a minimum 4 (4 items × 1 point) to a maximum of 28 (4 items × 7 points), giving a maximum total score of 84 (3 subscales × 28 points). The WAI‐SR correlates strongly with the WAI as well as with other measures of alliance, and is consistent with Bordin's (1979) model of the working alliance. The WAI‐SR has demonstrated good internal consistency and adequate convergent and predictive validity (Hatcher and Gillaspy 2006), and it has been demonstrated to have a clinically significant association with various measures of therapeutic outcome (Horvath *et al*. 2011, Munder *et al*. 2010).

### Assessment of stuttering severity, communication and quality of life (WASSP, OASES‐A)

The impact of stuttering severity, and stuttering‐related variables was measured using the Overall Assessment of the Speaker's Experience of Stuttering (OASES‐A) (Yaruss and Quesal 2006) and the Wright & Ayre Stuttering Self‐Rating Profile (WASSP) (Wright and Ayre 2000). In the WASSP, 26 questions are posed across five domains: (1) stuttering behaviours, (2) thoughts, (3) feelings about stuttering, (4) avoidance and (5) disadvantages due to stuttering. Internal reliability has been reported to be satisfactory (Wright and Ayre 2000). The OASES‐A consists of 100 items organized into four sections: (1) general information about stuttering and self‐awareness of stuttering behaviours, (2) affective, behavioural and cognitive reactions to stuttering, (3) communication difficulties in daily situations and (4) impact of stuttering on quality of life (Yaruss 2010, Yaruss and Quesal 2006). Each item is scored on a Likert scale ranging from 1 to 5, with higher scores indicating a greater degree of negative impact associated with stuttering. OASES‐A has demonstrated good test–retest reliability (*r* = .90–.97) and concurrent validity (*r* = .68–.93). Cronbach's alpha coefficient, calculated independently for each of the four sections of the instrument, revealed very strong internal reliability (*r* = .92–.97) (Yaruss and Quesal 2006). The Norwegian norms of the OASES‐A (Nordbø *et al*. 2018) were included to allow a comparison of the five impact scores (overall score and four subscores) with the scores from the present study.

### Assessment of the concept of motivation (CPST‐E)

In this study, an Extended version of the Client Preferences for Stuttering Treatment (CPST‐E) (McCauley and Guitar 2010) was completed by participants during the first pre‐treatment session only. The original CPST includes brief items regarding therapy goals, the individual's priorities with regard to stuttering, ease of participation in different speaking situations and a sense of control. Items are rated on a Likert scale rating from 1 (not at all important) up to 5 (very important).

The CPST‐E adds two further sections. One section includes questions regarding personal characteristics, including those related to the client's motivation based on Seo *et al*.’s (2010) ‘work motivation’ model: how persistent the client is in general, how motivated they are to work actively with their stuttering, how much time they are willing to set aside for independent training, how much help and support they expect during the therapy period, and their anticipations of the outcome. All the quantitative items are measured on a Likert scale, ranging from 1 (not at all) to 5 (completely). The second part is based on qualitative information and contains open text units where clients are required to write down their own goals and wishes for the therapy. The data set in the present study uses only the quantitative measures from the CPST‐E.

### Assessment of anxiety and depression (HADS)

A number of instruments are available to assess aspects related to fear of negative evaluation and anxiety. The Hospital Anxiety and Depression Scale (HADS) is a screening tool for screening both anxiety (HADS‐A) and depression (HADS‐D) (Zigmond and Snaith 1983). The internal consistency of the HADS‐A and HADS‐D showed coefficient alphas of .89 and .86 respectively, and has been found to be excellent in samples from general practice (Olssøn *et al*. 2005). The HADS is a self‐administered scale consisting of 14 items split across anxiety and depression subscales, each with a four‐point ordinal response format (e.g. ‘not at all’, ‘occasionally’, ‘quite often’ or ‘very often’). For this study, the first line in the introduction to the form was removed as it included the word ‘hospital’, which was inappropriate for our setting.

### Statistical analyses

The current study used a multiple single case design, with quantitative data collected at different time points: pre‐treatment (CPST‐E, OASES‐A, WASSP, HADS), after the second treatment session (WAI‐SR, T1), the fourth treatment session (WAI‐SR, T2), and at 1 (WAI‐SR, T3) and 6 months post‐treatment (OASES‐A, WASSP, HADS). Quantitative data were analyzed using IBM SPSS Statistics, version 25. Clinical and demographic data are presented as percentage and frequency (*n*). Norms of the OASES‐A, and subscores of the WAI‐SR (bond, goal and task) are presented as means (M) with corresponding standard deviations (SD).

The means and SD of the WAI‐SR were examined. Normality was assessed by obtaining skewness and kurtosis values, and descriptive statistics were calculated. Associations were calculated using Pearson's correlation coefficient (*r*), exploring the strength of the relationships between subscales and total scores of the WAI‐SR, and pre‐ and post‐intervention results of the OASES‐A, WASSP and HADS. Associations were also calculated between the WAI‐SR and the section of the CPST‐E that covers aspects related to the client's motivation for treatment, and expectations regarding support and treatment outcome. Treatment outcomes were measured using the relative delta scores (Δ) on OASES‐A, WASSP and HADS. The delta scores indicate change between two scores, given as a percentage (A ‐ B/A × 100). Level of significance was set to *p* < .05.

Multiple linear regression analyses were used to assess whether levels of motivation and willingness to set aside time for self‐training could predict perceived strength and quality of the relationship between client and clinician (WAI‐SR subscale *task*) and treatment outcome (OASES‐A). We wanted to control for the possible effect of initial stuttering and general distress, which could be associated with coping and treatment outcome, and test whether levels of anxiety (HADS‐A) and total impact scores (OASES‐A) were influencing the variables. The regression procedure consisted of two steps in order to control for the effect of each included variable, using forced entry. Preliminary analyses were conducted to test assumptions of normality, linearity, and multicollinearity. Statistical assumptions for the linear regression models were adequately met.

In order to compare subgroups with lower and higher scores on the measure of working alliance, the variable was dichotomized according to the WAI‐SR total score median value (50 percentiles), that is, ≥ 25 and < 25, leaving nine participants in each group. The criteria for cut‐off was set as a median because of the small sample size. For between‐groups comparisons, the Mann‐Whitney *U*‐test was used. Associations were calculated using correlation analyses (Spearman's rho), exploring the strength of the relationships between high or low WAI‐SR mean subscale scores (goal at T1 and task at T1), and delta total scores on OASES‐A, WASSP and HADS (total and subscales).

### Ethical considerations

Ethical approval was gained from the Regional Committee for Medical Research Ethics (2015/1275), and all participants provided written consent before participating in the study. All data were de‐identified.

## Results

Based on the WAI‐SR, we examined whether the therapeutic alliance correlated across the *goal*, *task* and *bond* subscales, and whether the working alliance was decreasing or increasing in quality throughout the treatment period. Thereafter, the relationship between the working alliance (as measured by the WAI‐SR), the motivation and willingness to set aside time for training (measured by the CPST‐E), and perceived improvements in communication, social activity, emotional restructuring and life‐quality (measured by the OASES‐A, WASSP and HADS) were explored.

### Goal, task and bond: total and mean scores on the WAI‐SR

**Figure 1:**
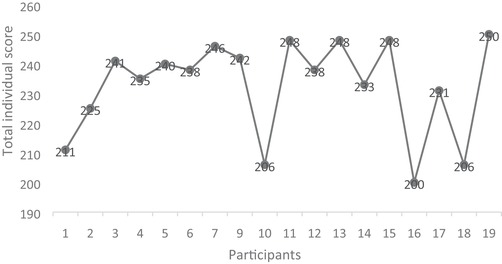
Summary of WAI‐SR total individual scores on goal, task and bond.

At an individual level, the variance of the total score in the present study ranges from 200 to 250 (T1 + T2 + T3 summarized) (figure 1).

**Figure 2:**
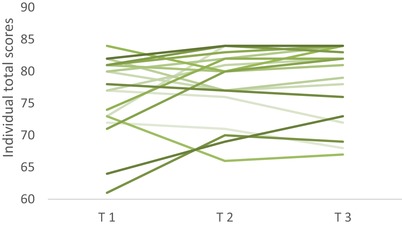
Results WAI‐SR individual total scores throughout T1, T2 and T3. [Color figure can be viewed at wileyonlinelibrary.com]

The variance between the individual total scores (goal, task and bond combined) ranged from 61 to 84 throughout T1, T2 and T3 (figure 2).

The mean subscale scores ranged from 24.5 (24.5/4 = 6.13) at the lowest (subscale task at T1) to 26.7 (26.7/4 = 6.68) at the highest (subscale bond at both T2 and T3). As seen in table 3, the quality of the working alliance seemed to be high throughout the study period. This may indicate an initial strong therapeutic alliance between the participants and the SLT, maintained throughout the treatment period at both the individual and group level. On a group level, the quality of the alliance increased during the period of treatment, but not significantly.

**Table 3:** Sample means (M) on the WAI‐SR *bond*, *goal* and *task* subscales

	T1		T2		T3		Total	
Subscales	M	SD	M	SD	M	SD	M	SD
Bond	26.3	2.52	26.7	2.59	26.7	1.87	79.7	6.52
Goal	25.4	2.45	26.4	1.85	26.6	1.87	78.7	5.51
Task	24.5	3.13	24.8	2.31	25.2	3.03	74.5	7.12
Total (maximum 84)	76.2	6.33	77.9	5.59	78.4	6.07		

Note: SD, standard deviation.

The highest variability between minimum and maximum mean scores was at the first evaluation (T1), and the greatest variability throughout the three data points T1, T2 and T3 is seen on the *task* subscale. Furthermore, aspects related to the *task* subscale in the WAI‐SR show the lowest scores, indicating that task‐related items may be the most sensitive elements in the working alliance (table 3). Of the three subscales, the *bond* subscale had the most stable scores.

### Associations between WAI‐SR and the concept of motivation

Associations between the working alliance and the concept of motivation were identified. The relationships between variables related to the clients’ concept of motivation, measured by the CPST‐E and the working alliance evaluated at T1, demonstrated a strong correlation, especially between the WAI‐SR *task* subscale and motivation (*r*(16) = .71, *p* = .001), time self‐training (*r*(16) = .76, *p* < .001), persistence (*r*(16) = .63, *p* = .005), expectation outcome (*r*(16) = .51, *p* = .031), and expectation support (*r*(16) = .60, *p* = .008), respectively. Inspired by the work of Seo *et al*. (2010), the three variables motivation, willingness to set aside time for training and the person's persistence were then combined into a single concept of motivation. The association between the WAI‐SR *task* subscale and the concept of clients’ motivation remained strong (*r*(16) = .781, *p* < .001).

To examine further the influence of motivation on the continuous WAI‐SR *task* subscale, a regression analysis was carried out to investigate the potential impact of motivation, and the presence of background variables (general distress and impact of stuttering). In order to control for the effect of each included variable, the regression procedure consisted of two steps. In step 1, the participants’ level of anxiety (HADS‐A) was entered. In step 2, self‐reported total impact score on the OASES‐A was entered. As can be seen in table 4, the influence of motivation was found to be statistically significant (*F*(3, 14) = .785, *p* = .002).

**Table 4:** Regression analysis evaluating the impact of motivation on the strength and quality of the relationship between client and clinician (WAI‐SR, *task* subscale), while controlling for initial levels of anxiety (HADS‐A) and stuttering impact (OASES‐A)

	B	(SE B)	Beta	*t*	*p*
HADS‐A (Anxiety)	−.014	(.158)	−.018	−.087	.932
Total impact score OASES‐A	−.009	(.012)	−.172	−.748	.467
Motivation	2.356	(.628)	.785	3.749	**.002**

Note: Data are presented as unstandardized coefficients (B), standard error of the unstandardized coefficient (SE B), standardized coefficient (Beta), *t*‐value (*t*) and *p*‐value (*p*).

### Associations between the concept of motivation and the treatment outcome

The same combined concept of motivation was associated with delta scores on the OASES‐A total scores, and showed a significant association: *r*(16) = .508, *p* = .031. The associations were also significant on the delta scores on two OASES‐A subscales: ‘Quality of Life’: *r*(16) = .539, *p* = .021; and ‘Communication in Daily Situations’: *r*(16) = .576, *p* = .012.

The relationships between the variable ‘Time set aside for training’ and the treatment outcome, measured by the relative delta scores on the WASSP and the OASES‐A, demonstrated significant correlations (WASSP: *r*(16) = .510, *p* = .031; OASES‐A: *r*(16) = .578, *p* = .012). The associations were also significant on three OASES‐A's subscales: ‘Your reactions to stuttering’, *r*(16) = .484, *p* = .042; ‘Communication in Daily Situations’: *r*(16) = .534, *p* = .023; and ‘Quality of Life’: *r*(16) = .578, *p* = .012.

To explore further the influence of ‘Time set aside for training’ on the outcome variable OASES‐A, we performed another regression analysis, following a similar procedure to that described above. In step 1, perceived anxiety (HADS‐A) was entered in order to control for this variable. In step 2, self‐reported total impact scores on the OASES‐A were entered. As seen in table 5, the results indicated that time set aside for training significantly explained treatment outcomes (*F*(3, 14) = .660, *p* = .011).

**Table 5:** Regression analysis evaluating the impact of ‘Time set aside for training’, while controlling for initial levels of anxiety (HADS‐A) and stuttering impact (OASES‐A) on treatment outcomes

	B	(SE B)	Beta	*t*	*p*
HADS‐A (Anxiety)	.406	(.590)	.166	.688	.503
Total impact score OASES‐A	−.042	(.040)	−.254	−1.046	.313
Time set aside for training	6.671	(2.266)	.660	2.944	**.011**

Note: Data are presented as unstandardized coefficients (B), standard error of the unstandardized coefficient (SE B), standardized coefficient (Beta), *t*‐value (*t*) and *p*‐value (*p*).

### Associations between the WAI‐SR and treatment outcome

Relationships were identified between the *task* and *goal* subscales (WAI‐SR), and the reduction of stuttering severity and anxiety. At a group level, results indicated a strong correlation between the quality of the working alliance (total score), and several outcome variables. Only one significant relation between the *bond* subscale and treatment outcome (WASSP‐Disadvantages) was found (*r*(16) = .507, *p* = .032). Significant correlations were found most notably on WAI‐SR items related to either *task* or *goal*. Based on the relative delta scores, several significant correlation between the *task* subscale and some relevant outcome variables (measured by the OASES‐A, WASSP and HADS) were found: HADS‐Anxiety: *r*(16) = .515, *p* = .041; OASES‐A‐total scores: *r*(16) = .541, *p* = .020; and WASSP‐total scores: *r*(16) = .495, *p* = .037. A few significant relationships between the *goal* subscale and outcome variables were also found: OASES‐A ‘Communication in Daily Situations’: *r*(16) = .608, *p* = .007; and WASSP‐Disadvantages: *r*(16) = .618, *p* = .006.

At a group level, all the presented relationships between the WAI‐SR and the outcome variables demonstrated a linear trend; the higher quality of the working alliance, the higher delta scores measured by the OASES‐A, WASSP and HADS‐A.

### Associations between ‘lower’ or ‘higher’ WAI‐SR, and treatment outcome

Finally, we selected one more variable that seemed clinically interesting in association between working alliance and outcomes. Several significant associations were noted between the quality of the working alliance and treatment outcome, when the working alliance was classified as ‘lower’ or ‘higher’ according to the WAI‐SR median values. Similar to other analyses in this study, the treatment outcome was measured based on relative delta scores on the OASES‐A, WASSP and HADS. The variables in the relationship between the working alliance and treatment outcomes demonstrated a similar trend. Thus, when the values related to the quality of the working alliance were considered as high, significant scores associated with positive treatment outcomes in terms of improved communication, reductions in anxiety, stuttering severity and avoidance behaviour were found, as presented in table 6.

**Table 6:** Correlations ‘lower’ or ‘higher’ WAI‐SR scores on the *goal* and *task* subscales, and treatment outcome

	WAI‐SR subscale Goal			WAI‐SR subscale Task		
	≤25	>25	*p*	≤25	>25	*p*
Δ OASES‐A (Total)	7.4(3.2‐13.9)	16.6(12.0‐31.8)	.024*	10.9(3.2‐13.1)	17.1(12.6‐31.8)	.012*
Δ OASES‐A (Communication)	2.1(−9.1‐8.3)	13.0(4.1‐22.5)	.031*	2.6(−5.7‐9.0)	13.0(0.6‐22.5)	.102
Δ WASSP‐Stuttering	13.0(9.0‐23.8)	26.4(18.2‐44.6)	.047*	17.6(7.2‐23.8)	30.1(14.9‐44.6)	.021*
Δ WASSP‐Thought	0.0(0.0‐21.0)	25.0(12.8‐39.4)	.031*	13.3(0.0‐34.1)	20.0(0.0‐29.2)	.653
Δ WASSP‐Avoidance	15.4(5.6‐26.1)	36.4(0.0‐46.8)	.248	12.5(0.0‐26.1)	40.9(13.2‐46.8)	.037*
Δ HADS‐Anxiety	14.3(‐9.1‐22.2)	40.0(11.1‐70.8)	.039*	7.1(−8.6‐22.2)	42.2(21.1‐72.9)	.010*

Note: Data are presented as median, interquartile range and *p*‐value (^*^ *p* < .05).

## Discussion

Although the present study forms part of a larger treatment study that takes stuttering *management* as its primary focus, we acknowledge that other factors may also influence treatment outcomes. Its aim was to investigate the quality of the working alliance between persons who stutter (PWS) and their SLT, and to investigate possible correlations between the working alliance, the concept of client motivation and management outcomes. Although causal relationships cannot easily be determined, we have succeeded in measuring the working alliance and relevant variables related to client motivation and management outcomes. The study confirms that the working alliance between an SLT and a PWS is important and is providing support for other studies in this field (Manning 2010b, Plexico *et al*. 2005, 2010). Based on Bordin's model, which identifies three dimensions contributing to the working alliance, our findings indicate that the dimensions of *task* and *goal* in the working alliance were particularly relevant.

### Importance of the working alliance and mutual agreement of tasks and goals

The results described above indicate that the quality of the working alliance and, in particular, variables related to mutual agreement of therapy tasks and goals, are relevant for treatment outcomes in stuttering treatment. As highlighted by, among others, Manning (2010a) and Ward (2018), goal‐directed treatment requires high clinical competence and flexible treatment procedures. Referring to the common factors model, Manning (2010a) also points out that ‘a therapeutic change is likely to be more successful if the clients and clinicians experience a therapeutic alliance that reflects a similar theoretical and practical perspective about the nature of the journey’ (314). However, although clinical decision‐making involving mutual agreement of goals and tasks demonstrated the strongest correlations and may be the most important factors, the role that may be played by the bond between the client and clinician should be acknowledged. In the present study, although only one significant relation between bond and outcome was found, the scores on the *bond* subscale remained stable throughout the treatment period. It is possible that this stable, underlying bond, perhaps associated with clinician and client's characteristics such as being present, receptive, genuine and committed, in which, according to Miciak *et al*. (2018), represent the intentions and attitudes in the clinical interaction and are needed for the physiotherapist and client to ‘*be’* in a therapeutic relationship. To some extent, the bond may provide the foundation for mutual agreement of tasks and goals, which is in accordance with Wampold's (2015) Contextual Model. This would correspond well with the work of multiple researchers who have highlighted the importance of clinician interpersonal style or quality in therapy (Ackerman and Hilsenroth 2003, Manning 2010a, 2010b, Nissen‐Lie *et al*. 2013, Oddli and Halvorsen 2014, Van Riper 1973), suggesting that these characteristics contribute to a bond between the SLT and the client, and which needs to be created initially in the therapy process (Wampold 2015).

All the ingredients in the working alliance are of importance. Nevertheless, based on the findings of the present study, it seems that the specific clinician characteristics that may contribute to the ‘bond’ are not as great an influence on the ‘principles of change’ as those related to mutual agreement regarding tasks and goals in therapy for adults who stutter. It is interesting to consider whether stuttering therapy may differ from psychotherapy in this respect. The psychotherapy research indicates that the effects of psychotherapy are primarily due to common factors in therapy, with factors common to many treatments explaining a larger percentage of the variance in treatment outcome than the specific ingredients associated with different treatment protocols (Wampold 2015). Owing to the nature of SLT, it is possible that it may typically be more task‐based than psychotherapy and, therefore, as Wampold considered (personal communication with the first author, 13 August 2018), ‘it makes sense that in the context of speech therapy, the bond would be less important than in psychotherapy, where the focus is often on an “inner” experience’.

### Relationship between the working alliance and the client's motivation

The client's personal characteristics include, among others, features such as motivation, persistence, the willingness to set aside time for training, the individual's expectation of a positive outcome, and the individual's expectations of support during the therapeutic process. In the present study, each of these variables, and particularly the client's self‐reported motivation and willingness to set aside time for training, was associated with the working alliance. The strongest correlations were with the *task* subscale, measured using the WAI‐SR. Within our study, we defined the concept of motivation as a combination of subjective motivation, willingness to set aside time for training and expectation of a positive outcome (Seo *et al*. 2010). This concept of motivation was subsequently identified as a significant predictor for a positive outcome. These findings confirm those of Herder *et al*. (2006) who found significant associations (*p* < .01) between the *task* subscale and the clients motivation. This highlights the potential interaction of meaningful tasks and client motivation for achieving positive change. Our finding that the client's expectations of support are relevant corroborates the work of Manning (2010b) and Plexico *et al*. (2005, 2010), who state that the degree of support and help a person who stutters can expect may influence treatment outcomes.

### Significant relationship between the working alliance and treatment outcomes

The relationship between the quality of the working alliance as perceived by the client early in the treatment (T1), and treatment outcomes was investigated. Several variables in this relationship demonstrated a linear, positive trend or tendency. Thus, when the values reflecting the quality of the working alliance were high (indicating a positive working alliance), scores reflecting treatment outcomes associated with communication and social activity indicated positive change. In more detail, a significant positive relationship was identified between the *goals* and *tasks* subscales of the WAI‐SR and reductions in anxiety, stuttering severity and avoidance behaviour.

Based on these findings, it was considered clinically meaningful to categorize the participants into two different groups; those with relatively lower scores (≤ 25) on the *task* and *goals* subscales, and those with higher scores (> 25). It is important to point out that the scores are relative and, although scores < 25 do not indicate a poor working alliance, these fairly subtle differences in the experience of the working alliance appeared to have a measurable impact on treatment outcomes. By analysing these two groups, we were able to demonstrate the tendency for those regarding the working alliance most positively early in treatment (at T1) to experience the most positive outcomes 6 months post‐therapy. As recommended by Wampold (2015) and Del Re *et al*. (2012), among others, this suggests there is considerable therapeutic potential in clinicians devoting time to developing a positive working alliance. This finding also suggests the direction of the ‘collaborative journey’, and the idea that the client's experience of the working alliance at an early stage in the therapy process is associated with treatment outcomes. A key question is whether a client who regards the quality of the working alliance as very high at the beginning of the treatment process is, therefore, better able to accurately predict a more successful treatment outcome. Or the converse; if a client perceives the working alliance to be ineffective or does not trust or feel confident with the SLT, this may directly influence the treatment outcome. If this is the case, such concerns might suggest that the client would benefit from reconsidering the choice of clinician.

The present study has highlighted associations between variables related to the working alliance and treatment outcomes and demonstrated that the working alliance is a critical component for successful treatment for adults who stutter. These findings provide support for the position of StutterTalk©, who advise individuals who stutter to consider trying multiple clinicians in order to find a positive therapeutic relationship. Although access to SLTs may be limited by the individual's location, local provision of services, and access to such services, we strongly support the basic tenet that the working alliance matters, and that a stable and trusting client‐clinician relationship is a central factor within treatment for stuttering.

### Strengths and limitations

The multiple single case design together with combining the WAI‐SR with stuttering measures, has allowed the consideration of interactions both between and within individual factors. As with many case studies, the greatest limitation of our study remains the small size of the sample. Bearing this in mind together with the fact that the sample is taken from a treatment study rather than day‐to‐day working alliances within a clinic setting, the preliminary results should be interpreted within this specific context.

We report only on subjective measures in this study. Although such measures and evaluations can be regarded as very useful in the evaluation process, we highlight the need for researchers and clinicians to be aware of the variability and differing reliability in clients’ awareness and perspectives, and that validated measurements should therefore be used in a careful and transparent manner. We suggest that a combination of subjective self‐evaluation, plus objective professional measurement might represent the optimal client‐centred, outcome‐focused result in clinical research.

A potential weakness of the study is the risk of conflating causation with correlation. The working alliance constructs are particularly relevant here, as it is not clear whether a better outcome in treatment leads to a stronger working alliance or vice versa. In other words, even though we have implicated a possible direction in some relationships, the causality of the relationship underpinning the change in direction remains unclear. The client's satisfaction with the alliance would need to be measured for an extended period in order to draw more certain conclusions.

A further potential weakness is the participants’ relationship with the researching clinician (lead author) who implemented the treatment. Although the clinician was blinded to the evaluations during the treatment period, there is a risk that participants may have underreported negative experiences and over‐reported positive experiences to protect their relationship with the clinician. However, measures were taken to reduce this risk (see the methods section) and, to our knowledge, participants’ evaluations were honest, authentic and representative. Despite these caveats, we hope, that the results presented will encourage others to replicate and expand the research with larger and more heterogeneous samples to establish the robustness of the present findings.

## Conclusions and implications

SLTs need to be aware of the importance of the working alliance in stuttering treatment. Our findings support the use of the WAI‐SR as a useful tool for evaluating elements of this relationship, specifically shared understanding of treatments goals, agreement regarding treatment tasks, and the bond between the parties. It is possible that the working alliance, in particular, the shared understanding of goals and agreement on tasks, training and activities relevant to these goals, might be among the most critical elements for successful treatment. Clinicians have previously recommended the use of measurements to examine the quality of the therapeutic alliance as an essential component of clinical work and research. Incorporating such evaluations at an early stage of treatment could permit SLTs and clients to identify and repair challenges should they arise.

Pre‐existing evidence suggests that clinicians who are better able to form alliances with clients, have better outcomes with their clients than other clinicians. Clients may influence the alliance and outcomes in many ways, including through their own motivation, but the present research suggests that clinician characteristics are important, too, and that SLTs who are more responsive to their clients’ individual characteristics, facilitate positive treatment outcomes.

The client's motivation for treatment, and mutual agreement regarding meaningful tasks for achieving the desired goals or changes, may become important predictors for successful therapy outcome. This multiplicity of factors associated with treatment outcomes corresponds well with several of the models described in the introduction, including the Common Change Principles, and the Contextual Model. Interestingly, they do not correspond as well with the estimations of Lambert's (2013) pie chart, as it seems that tasks or techniques may play a larger role within stuttering therapy outcomes than Lambert's model predicts. Future research inspired by these findings could include further investigation of the contribution of clinician characteristics to the working alliance and to treatment outcomes in stuttering therapy.

## Note

1 Statped is a national service for special needs education, defined for different core areas, including services for speech and language disorders.
